# Rapid self-recognition ability in the cleaner fish

**DOI:** 10.1038/s41598-025-25837-0

**Published:** 2025-11-25

**Authors:** Shumpei Sogawa, Taiga Kobayashi, Redouan Bshary, Will Sowersby, Satoshi Awata, Naoki Kubo, Yuta Nakai, Masanori Kohda

**Affiliations:** 1 Laboratory of Animal Sociology, Department of Biology, Graduate School of Sciences, Osaka Metropolitan University, Osaka, Japan; 2 Institute of Zoology, University of Neuchâtel, Neuchâtel, Switzerland; 3 Department of Energy, Environment and Climate Action, Arthur Rylah Institute for Environmental Research, Victoria, Australia

**Keywords:** Evolution, Psychology, Zoology

## Abstract

Whether animals are self-aware has important implications for our approaches to both animal cognition and animal welfare. A landmark moment in animal cognition research was when great apes passed the mark-test and demonstrated mirror self-recognition (MSR). Animals that pass the mark-test are capable of visually self-recognising and considered to be self-aware. Other taxa, including a fish, the cleaner wrasse (cleaner fish: *Labroides dimidiatus*) have also now passed the mark-test, forcing a rethink of the mental and neurological requirements for MSR. Previous research has largely focused on which species can pass the mark-test, rather than the processes underlying MSR. Here, we marked mirror-naïve cleaner fish with an ecologically relevant mark resembling an ectoparasite and then undertook detailed behavioural observations after exposure to a mirror. We found that cleaner fish achieve MSR rapidly, implying self-awareness prior to mirror exposure. By observing the exact timing of MSR in individuals, we could also report previously undocumented differences in pre- and post-MSR behaviours, including post-MSR exploratory behaviour of the mirror’s reflective properties. We find remarkable parallels between the processing of MSR in humans and cleaner fish, suggesting that some aspects of self-awareness are conserved across animal taxa.

**Supplementary Information:**

The online version contains supplementary material available at 10.1038/s41598-025-25837-0.

## Introduction

Whether animals are self-aware has remained a popular yet controversial topic in both philosophy and the natural sciences. Although there are several definitions of the term self-awareness, we primarily use the term as per Kohda et al.^1^ but see Morin^2^. Two alternative hypotheses have been proposed to explain the possible evolution of self-awareness in vertebrate animals. One is a “Big Bang” hypothesis, which posits that true self-awareness evolved only once in the common ancestor of the great apes^3^. This first hypothesis was formulated based on observations by Gallup that chimpanzees, but not monkeys like Macaques, can pass the mark-test^3,4^. The mark-test is a standard approach for testing self-awareness in animals, whereby a mark is placed on the body that is only visible in a mirror reflection. Animals that subsequently try to interact with the mark during mirror exposure are considered capable of mirror self-recognition (MSR). In addition to chimpanzees, MSR has now been documented in several other vertebrate taxa, including the bottlenose dolphin, Asian elephant, Eurasian magpie, Indian house crow and the cleaner fish^1,5,11,12,13^. However, these results remain controversial and are not always considered indicative of true self-awareness^4^. In contrast, the apparent presence of MSR in some species, but less sophisticated mirror interactions in others, led to the alternative “gradualist” hypothesis for describing the evolution of self-awareness in animals^14^. This second hypothesis considers a scale of simple to complex self-awareness, where the capacity to use a mirror as a tool precedes the ability to recognise the self in the mirror, which in turn is considered a less cognitively complex task than true MSR.

Both hypotheses were developed with the central premise that failure to pass the mark-test indicates no capacity for MSR and no or limited self-awareness. However, neither hypothesis considers the potential for false negatives to impact interpretations made from mark-tests [e.g^12,15,16^. or that detailed behavioural observations may reveal that many species do interact with mirrors in other ways, suggestive of a cognitive capacity for self-awareness. For example, some animals that fail the traditional mark-test still interact with the mirror and can exploit its reflective properties to their advantage, yet these abilities have either been considered unrelated to self-awareness^3,17^ or as examples of less complex forms of self-awareness^14^. What has not been previously considered is that animals that begin to use the mirror as a tool (e.g. to view objects only visible in the reflection) may have already achieved MSR. Indeed, MSR mark-test experiments are typically only conducted after several days or even weeks of mirror exposure [e.g^1,3,5,8,10,12,13,18^. During this period of extended mirror exposure, only broad behavioural observations have been made with the aim of recording self-directed behaviours, which have traditionally indicated that an animal is now ready to attempt the mark-test. Remarkably, the actual timing of MSR after mirror exposure still remains unknown, including whether animals have already established MSR prior to using the mirror as a tool. As such, detailed behavioural observations may reveal important information that could refute the two main hypotheses currently explaining the evolution of MSR and self-awareness in animals.

It is generally acknowledged that animals exhibit three key behavioural stages prior to passing the mark-test^1,3,5,6,8^. First, they display social behaviours towards the mirror, suggesting an initial interpretation of the reflection as an unknown conspecific. Second, they begin contingency-testing (C-testing) between their body movement and the movements in the mirror. Third, they exhibit self-directed behaviours, including examining otherwise invisible body parts in the mirror reflection. Only during this final third stage have mark-tests been conducted to confirm MSR in animals^1,3,5,6,8^. These three behavioural stages occur after mirror exposure and are documented across taxa (e.g. mammals; [3, 8], fish; [1, 5], birds; [19]) suggesting a possible common cognitive basis for MSR in vertebrates^1,5^. We predict that uncovering the exact timing of MSR will also disclose that these three stages occur distinctly, rather than overlap as is commonly assumed [e.g^3,5^. We suspect that when animals have demonstrated self-directed behaviours, it is logically inconsistent this will then overlap with any social or C-testing behaviours, because when animals recognise their own mirror image as the self, they are unlikely to continue either aggression or contingency behaviours toward their own reflection.

Mirror self-recognition has previously been demonstrated in the blue-streak cleaner wrasse (cleaner fish; *Labroides dimidiatus*) via the mark-test. Specifically, cleaner fish were observed attempting to remove, by scraping on substrate, a mark placed on an otherwise non-visible part of their throat^5,12^. Here, we investigate exactly when MSR occurs in this species by placing a mark on the throat of cleaner fish prior to any previous exposure to a mirror. This is a novel approach, which will provide us with the opportunity to identify the exact timing of MSR. As stated, previous experiments have relied on multiple days or weeks of mirror exposure before placement of the mark and attempting the mark-test. By placing the mark before mirror exposure, we can identify exactly when MSR occurs by recording the first throat scraping behaviour after mirror presentation, rather than waiting to attempt the mark-test only after observing C-testing behaviours, potentially several days after mirror exposure.

To date, very limited information is available on the timing of MSR, largely coming from studies on mirror-naïve human toddlers^20^ and a group of adults in Papua New Guinea^21^. Human MSR does occur within minutes after the first instance of being exposed to a mirror, which is unsurprising considering humans are self-aware with or without access to a mirror. Demonstrating that animals are self-aware presents greater challenges compared to humans, but mirrors and other images (e.g. photographs) can be utilised to demonstrate self-awareness in animals post, and importantly, prior to mirror exposure. Cleaner fish can recognise themselves in photographic images via self-face recognition, but only after MSR, suggesting that cleaner fish are inherently self-aware and capable of memorising the image of their own face^1^. Previous studies have assumed, without clear evidence, that MSR in animals requires extended exposure to a mirror [e.g^3,5,8,10^. However, if like humans, cleaner fish are capable of rapidly achieving MSR, which is only possible if they are already self-aware, then the mental process underpinning self-recognition are expected to be similar across vertebrate taxa. On the other hand, if MSR in cleaner fish is instead produced by some form of associative learning^4^, then MSR cannot occur rapidly.

The cleaner fish is a small fish that inhabits coral reefs and rocky shores within the tropical and sub-tropical Indo-Pacific. The species specializes in removing and consuming small ectoparasites from the bodies of various ‘client fish’. Fishes display a wide range of cognitive abilities^22^ and the cleaner fish is a well-researched model species for the study of fish cognition, demonstrating the strategic use of tactical deception^23^, transitive inference^24^, a strong ability to delay gratification^25^, key elements of theory of mind^26^ and MSR-capability^12^. In this study, we first assess how quickly cleaner fish can establish MSR. Second, we investigate why cleaner fish, and more broadly other animals, appear to display social and apparent C-testing behaviours in the days following exposure to a mirror^1,3,5,8,12^.

## Results and discussion

### Behavioural responses to mirror presentation

**Fig. 1:**
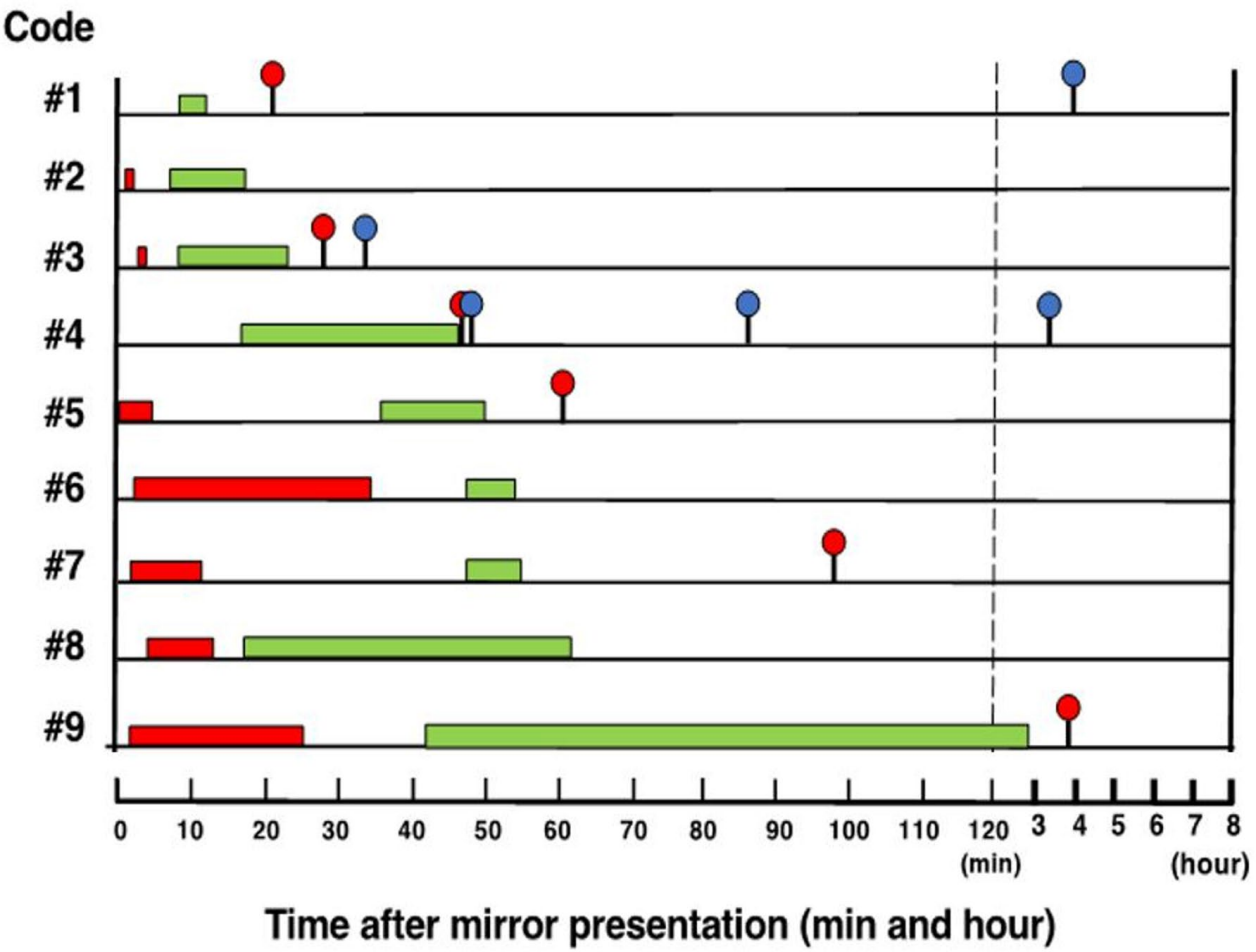
Timeline of distinct behavioural responses toward mirror self-image. Aggression stage (red bar), contingency-testing (C-testing) stage (green bar), first attempt at mark removal (red circle) and subsequent attempts at mark removal (blue circle) in individual cleaner fish (*n* = 9). Behavioural stages (aggression and C-testing) are defined as periods of time (minutes) and begin and end at the first and last observations of those behaviours in an individual, respectively.

Cleaner fish that were marked prior to mirror exposure were observed exhibiting two typical distinctive behavioural stages (also observed in other animal MSR studies; 3, 5, 10, 12) when first presented with a mirror. At the very start of mirror presentation, seven of the nine cleaner fish exhibited aggressive behaviours toward their mirror-image, ranging from between 31 and 1867 s (median of 513 s; Fig. 1). Aggressive behaviours then subsided, and fish were typically observed swimming at the opposite end of the tank to the mirror, but occasionally approaching and swimming away from the mirror, for a duration ranging from 168 to 2067 s (median 721 s, *n* = 7). Following this period, focal fish were observed performing distinctive behaviours toward their mirror image (Fig. 1, Table S1, Movie S1a, b, see Materials and Methods). Specifically, focal fish were observed approaching their reflection and appearing to gaze at the image, while momentarily leaning towards and/or aligning vertically with the mirror (Behaviours a and b in Table S1). Other behaviours included a gazing-type behaviour while rapidly swimming away from the mirror (Behaviours c and d). We regard these behaviours toward the mirror image as contingency-testing (C-testing: Movie S1 a, b), which were observed in all fish performed for a period ranging from 225 to 8419 s (median 829 s; C-testing stage; *n* = 9; Fig. 1). The amount of specific C-testing behaviours performed by individuals varied between 5 and 46 (median of 10; Table S1). Both aggression and C-testing behaviours were only performed for a clearly defined period of time after which they were not observed again during mirror exposure. As predicted, the two stages were also temporally distinct and separated by a period of non-aggression toward the mirror image (Fig. 1).

The first attempts by individual cleaner fish to remove the mark on their throat began after the last C-testing behaviour was observed (range = 2.0 to 54.7 min; Fig. 1). Six of the nine fish (67%) attempted to remove the coloured elastomer mark placed on their throat within two hours of mirror exposure. The first mark-scraping behaviours by these fish ranged between 15 and 3283 s (median 613 s) after the last respective observed C-testing behaviour (Fig. 1). Previous MSR studies have typically exposed animals to a mirror for days or weeks before attempting the mark-test, yet the passing ratio of the mark-test we implemented (i.e., placing the mark prior to mirror exposure) is comparable or higher than recorded in many other MSR capable taxa^8,10,27^. Our results clearly demonstrate that MSR in cleaner fish can occur remarkably rapidly (i.e., as quickly as 30 min after mirror exposure) and does not require extended prior exposure to a mirror. Our observations also confirmed that cleaner fish exhibit distinct and non-overlapping behavioural stages before establishing MSR.

### Behavioural responses post-MSR

We continued behavioural observations post-MSR, including for the three individuals that did not attempt to remove the mark. One of those individuals did subsequently display throat scraping behaviour, and the other two individuals displayed distinct aggression and C-testing behaviours, but not throat-scraping (Fig. 1).

**Fig. 2:**
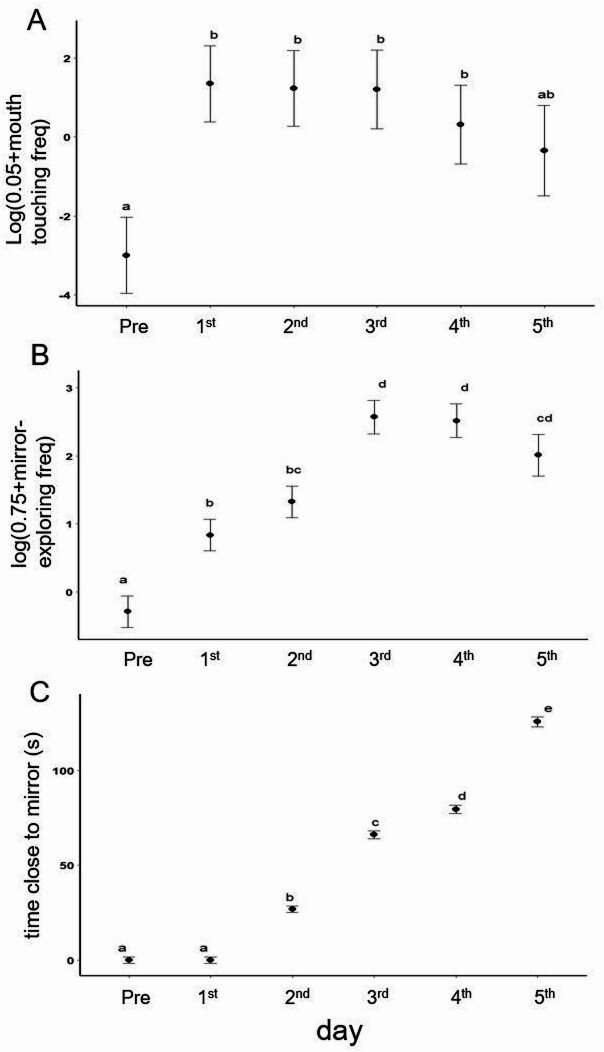
Linear model predictors (Tukey contrasts) on the frequency of different behaviours pre-MSR (before mark removal attempt) and on the following 5 days, showing mean +/-SD. (**A**) Frequency of the mouth touching the mirror (data log transformed); (**B**) frequency of ‘mirror-exploring’ behaviours (data log transformed); and (**C**) time within 5 cm of mirror. Letters above each plot indicate significance levels: a shared letter indicates lack of significant differences; and different letters indicate significant differences.

After attempting to remove the mark (i.e., post-MSR), cleaner fish behaviour shifted to resemble behavioural observations previously made on unmarked individuals exposed to a mirror [e.g.^5,12^]. For instance, cleaner fish were recorded mouth-touching the mirror surface, exhibiting atypical swimming patterns in front of the mirror, and spent more time within 5 cm distance of the mirror (Fig. 2, also previously described in Fig. 1 of^5^ and Fig. 4 of^12^). Knowledge of the exact timing when MSR occurred (at the individual level) allowed us to compare and interpret the context underlying these behaviours exhibited pre- and post-MSR, which has not been possible in previous studies^5^. We found post-MSR, cleaner fish behavioural patterns differed clearly in three distinct ways compared to pre-MSR. First, cleaner fish mouth-touched the mirror significantly more frequently (LMM, df = 5, F = 6.7, *p* = 0.0002, Tukey contrasts showing that pre-MSR frequency was lower than on each of the four following days with all *p* < 0.006; Fig. 2A). Second, cleaner fish exhibited significantly more atypical swimming patterns in front of the mirror, starting from day 2 post-MSR (Bayesian LMM, *df* = 5, χ^2^ = 155, *p* < 0.0001, Tukey contrast showing that pre-MSR and day 1 yielded less atypical swimming patters than all other days with all *p* < 0.0001, and further significantly increased in frequency between all following days; Fig. 2B). Third, compared to pre-MSR, cleaner fish spent significantly more time closer (< 5 cm) to the mirror surface (LMM, *df* = 5, F = 26.1, *p* < 0.0001, Tukey contrast between pre-MSR and all other days all *p* < 0.002, various differences between other days; see Fig. 2C). These behavioural patterns appear similar to observations made on unmarked cleaner fish that are exposed to a mirror for several days (see [5, 12]). In previous studies, clear fish being motionless or swimming slowly nearby the mirror surface had been interpreted as the individual observing their own image and classified as a self-directed behaviour^5^.

**Fig. 3:**
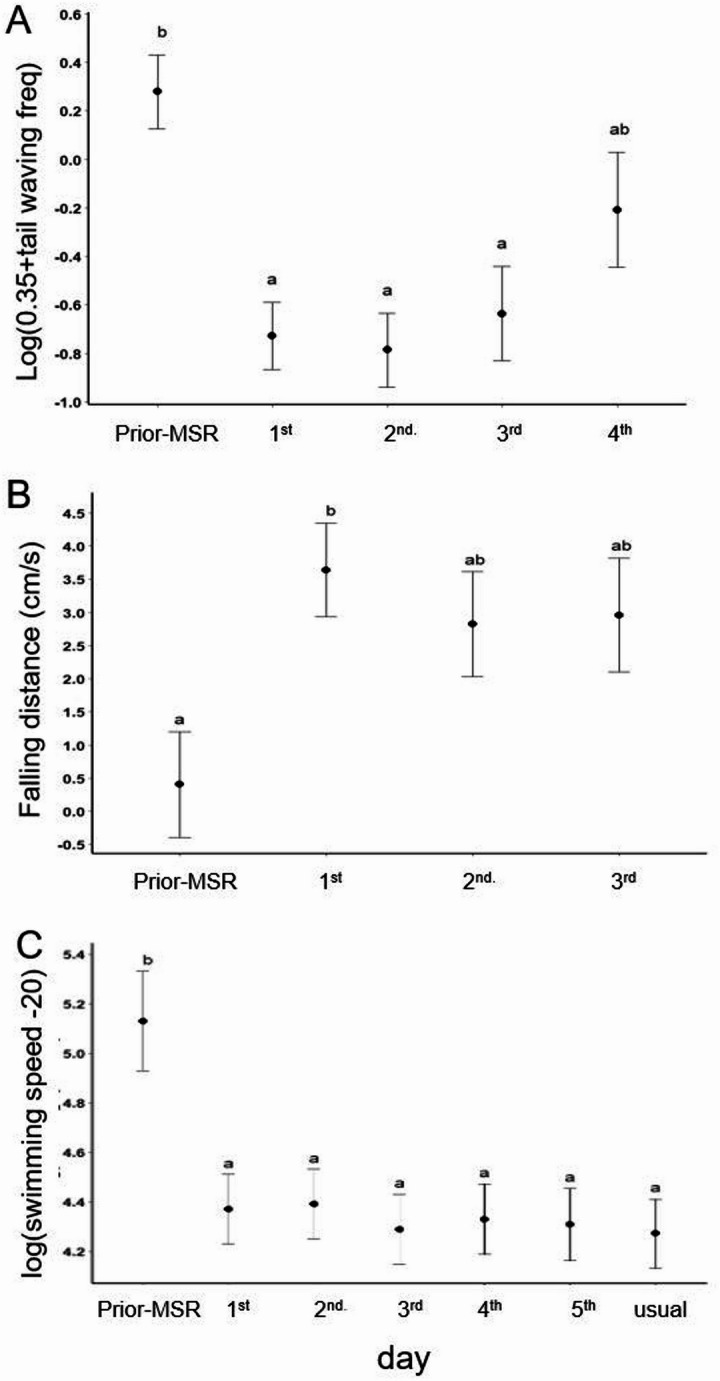
Comparison of means +/-SD of behaviours exhibited pre-MSR and post-MSR (day). (**A**) Mean frequency of caudal fin waving (linear mixed model [LMM], *df* = 4, χ^2^ = 48.85, *p* < 0.0001, *n* = 5 fish; Tukey HSD, a vs. b: *p* < 0.05; (**B**) mean distance sinking down mirror (LMM, *df* = 4, χ^2^ = 33.54, *p* < 0.0001, *n* = 5 fish; Tukey HSD, a vs. b: *p* < 0.01; and (**C**) mean swimming speed (LMM, *df* = 6, χ^2^ = 279.74, *p* < 0.0001, *n* = 7 fish; Tukey HSD, a vs. b: *p* < 0.001. Pre- and post-MSR swimming speed was also compared against swimming speed under non-experimental conditions.

We observed remarkable differences in pre- and post-MSR behavioural responses when cleaner fish touched the mirror with the mouth. First, the frequency of caudal fin waving was significantly higher during pre-MSR aggressive behaviour, compared to post-MSR mouth-touching behaviours (LMM, *df* = 4, χ^2^ = 48.85, *P* < 0.0001; Fig. 3A; Movie of aggression S2; mouth-touching S3). Second, pre-MSR aggression (e.g. biting and charging) was concentrated solely toward their own mirror image, whereas post-MSR mouth-touching behaviour involved comparatively lighter touching of the mirror surface and included simultaneously sinking along the mirror surface toward the bottom of the tank (sinking distance: *df* = 4, χ^2^ = 33.54, *p* < 0.0001; Fig. 3B; Movie S3). Third, swimming speeds pre-MSR were significantly faster compared to post-MSR, which in turn did not significantly differ from typical swimming speeds prior to mirror exposure (LMM, *df* = 6, χ^2^ = 279.74, *p* < 0.0001, Fig. 3C). Additionally, these patterns of behaviour post-MSR were consistent with a reanalysis in previous study^5^; Supplement Fig. S1). Overall, the behaviours displayed post-MSR suggested that fish were in a more relaxed physiological state, compared to pre-MSR (e.g., slower swimming speeds, less frequent fin movements, non-aggressive interactions with the mirror). Within the novel context of knowing the exact timing of MSR in focal individuals, we interpret the differences in pre- and post-MSR behaviour as cleaner fish identifying the mirror image as self post-MSR, while assuming the presence of a perceived unknown/rival conspecific and acting aggressively pre-MSRs^28,29^.

Excluding mouth-touching, we observed cleaner fish displaying five other distinctive yet atypical behaviours post-MSR (Table S2). These behaviours were typified by dynamic and quick motions, including rapid swimming along and against the mirror surface (> 10–30 cm; Movie S4, a, b). While quantifiably different to the behaviours displayed pre-MSR (e.g., C-testing behaviours; Movie S1 a, b) these post-MSR behaviours show striking similarities with behaviours described in previous MSR studies during days 1 to 5 of mirror exposure, prior to the mark-test (see^5,12)^. Importantly, the atypical behaviours here were: *i)* not observed in the absence of a mirror, *ii*) always oriented towards the mirror, *iii*) repeated, and *iv*) rapid or dynamic movements (Table S2). Previous studies have classified these atypical behaviours as C-testing^5,12^ however these descriptions were made without knowledge of the exact timing of individual MSR. In other words, interpretations made from previous MSR studies^5,12^ have been uninformed on the central aspect of passing the mark-test, i.e., the exact timing of individual MSR.

In fishes, C-testing behaviour is categorised by alignment of the body with the mirror reflection and direct gazing at their mirror image, even when swimming away from the mirror (Table S1, Movie S1 a, b). In contrast, post-MSR, mirror alignment and gazing toward the mirror image were rarely observed and cleaner fish instead displayed the described atypical behaviours (Table S2, Movie S4 a, b). In addition, we also reanalysed video-recordings made from a previous MSR cleaner fish study and confirmed that mirror alignment and mirror gazing behaviours were absent after one day of mirror exposure (see Table 2 in^12)^. The most parsimonious explanation for these behaviours is that pre-MSR behaviours directed toward the self-mirror image are C-testing, whereas behavioural activity post-MSR is exploring the causal inference of the mirror reflection, which we term ‘mirror-exploring behaviour’.

Further supporting our claims of mirror-exploring behaviours post-MSR is our remarkable observation of a previously undescribed behaviour in cleaner fish. Three cleaner fish were recorded on days three and four of mirror presentation picking up pieces of fresh shrimp from the tank substrate, lifting them up ~ 10–25 cm and dropping the pieces close to the mirror. The cleaner fish followed the sinking shrimp pieces down the mirror, mouth-touching the mirror surface, observing the sinking food in the mirror reflection (see Movie S5). Our interpretation is that they were testing the contingency of the moving material other than itself to explore the mirror property. Comparable behaviours have been observed in manta rays, which watch rising bubbles in mirror reflections^30^, and in bottlenose dolphins that produce and play with bubbles in front of the mirror^31^.

## General discussion

Mirror self-recognition studies have played a fundamental role in our understanding of animal cognition for over half a century. By making the most detailed behavioural observations of any MSR study to date and by developing a novel variant of the mark-test, we have observed for the first time exactly when individuals first achieve MSR. Our study addresses five principal issues that have until now remained unresolved in previous MSR studies. They are: (*i*) the functional roles of the multiple distinct behaviours animals exhibit toward their own mirror image; (*ii*) how MSR relates to an individual’s possession of a sense of self; (*iii*) the relationship between MSR and the ability to use mirror reflections as a tool; (*iv*) the cognitive ability and encephalization quotient requirements for MSR; and (*v*) the wide taxonomic distribution of MSR and self-awareness across the animal kingdom.

It had been assumed that MSR in non-human animals first required days or weeks of mirror exposure^1,3,5^. Our results represent a paradigm shift in our understanding of animal self-recognition, demonstrating that like humans, other animals are also capable of rapidly recognizing their own mirror image. We also show that pre- and post- MSR behaviours are significantly different, with pre-MSR behaviours shaped by aggression and examination of their own mirror image, whereas various post-MSR behaviours appear to explore the causal inference of the reflective properties of the mirror (point *i*). It is highly improbable that cleaner fish represent a unique example, but instead we propose that our results offer a broader advancement in our understanding of how MSR occurs in animals more generally. We predict that detailed re-examinations of the behaviours with a large enough mirror exhibited by other MSR capable species will also likely reveal much more rapid achievement of MSR than previous assumed. Moreover, we predict that other species will display comparable differences in pre- and post-MSR behaviours and in a shorter timeframe than previously reported.

Mirror-naïve humans quickly achieve MSR, implying self-awareness prior to encountering a mirror^20,21^. While this is an intuitive result in humans, demonstrating that animals are self-aware, experimentally and independent of any preconceived notions, has proven challenging. The rapid achievement of MSR by cleaner fish (point *ii*), combined with their ability to recognise the self in the mirror via C-testing^12,29^, strongly implies a pre-existing sense of self. The alternative explanation, which we consider improbable, is that cleaner fish rapidly develop or form an apparent self-awareness only after exposure to a mirror as a result of some kind of learning. We consider the observations of rapid MSR in cleaner fish as the first direct empirical evidence that non-human animals possess self-awareness prior to exposure to a mirror.

It is widely assumed that the ability to use mirrors as a tool for gaining information is not cognitively equivalent to recognizing the self in a mirror image, with many more examples of the former than the latter (e.g., Lesser ape^32^; African Grey parrot^33^; Pig^34^; Sea lion^35^; New Caledonian crow^36^; Keas and Cockatoo^37^; Eurasian jay^38^; Japanese monkey^39^; capuchin monkey^40^; two Macaca monkeys^41^, but see^15,39^. The assumption has been that MSR requires a relatively ‘sophisticated’ level of self-awareness, in contrast to the ability to recognise other objects in a mirror reflection (e.g.^33,35^). This assumption has been formed largely due to the strict interpretation that failure to pass the traditional mark-test means a lack of self-awareness. However, here we generate a new prediction (point *iii*) that MSR occurs prior to the performance of mirror-exploring behaviours testing the causal inference of the mirror’s reflective properties, which in previous studies have been misidentified as C-testing. Several other species, including domestic pigs, rhesus monkeys, manta rays, corvids and domestic fowl, have all failed the mark-test but do perform C-testing behaviours and have demonstrated mirror tool use [e.g.^16,19,30,34,42^. Our results suggest however that these animals do have a pre-existing self-awareness and have likely already achieved true MSR via C-testing behaviours before then displaying mirror-exploring behaviours and using the mirror as a tool. We interpret the failure of these species to pass the mark-test as a false negative due to inherent species-specific methodological constraints, rather than a lack of ability to self-recognise or having no or ‘simple’ self-awareness^5,12,15,16^. We predict that detailed behavioural observations and/or re-examination of previous video recordings, like those conducted in our study, may demonstrate the establishment of MSR via key behavioural shifts in these species previously considered not capable of MSR.

The capacity to recognise the self in a mirror reflection was first expanded beyond humans^42–44^ to the great apes but initially did not include other species (points *iv* and *v*^3,18,27)^ . At the time it was plausibly assumed that MSR is an advanced cognitive capacity occurring within a correspondingly narrow species distribution. When the taxonomic scope of the mark-test did extend beyond the great apes, the species tested for MSR capacity were still selected based on their advanced cognitive capacity and/or high encephalization quotient (e.g., dolphin^31^; Asian elephant^8,45^; magpie^10^; giant manta ray^30)^. Several clear demonstrations of MSR in the cleaner fish ([1, 5, 12, 13] and present study) questions the traditional rationale for selecting species to undertake the mark-test, suggesting that neither relatively advanced cognition nor encephalization are key to MSR ability.

In summary, the ability of cleaner fish to rapidly demonstrate MSR implies they have an inherent self-awareness. Traditionally it has been assumed that failure to pass the mark-test demonstrated a lack of self-awareness or that self-awareness occurs in a gradient across taxa from simple to more complex. Our results do not support self-awareness evolving once in the hominid great apes nor that it has evolved incrementally across vertebrates. We instead propose that MSR is a skill, one that is facilitated by being self-aware. More broadly, our results suggest that self-awareness evolved at a minimum with the bony fishes (450 Mya)^46^ and is likely widespread across vertebrates. Our proposal will have wide implications across a range of fields, including philosophy, neuroscience, psychology and evolutionary biology.

## Materials and methods

### Study system and animal husbandry

All cleaner fish were wild caught and obtained from a commercial aquarium retailer. Cleaner fish are protogynous hermaphrodites, changing sex from female to male, with a polygynous harem mating system^47,48^. In our experiment we used nine mirror naïve cleaner fish (56–72 mm TL) that would be female according to body length. Fish were kept visually isolated from each other in separate experimental tanks (45 cm x 30 cm x 28 cm; water 26 °C; 12 h light/dark regime). A small piece of diced fresh shrimp hung on a fine wire and commercial fish food (Tetramin) were provided between 8:40 and 13:00 each day. All fish were kept in experimental tanks for a minimum of two weeks prior to commencement of experiments.

### Previous descriptions of mirror related behaviours pre-mark-test

Kohda and colleagues have previously described three distinct behavioural stages in cleaner fish after exposure to a mirror (Fig. 1A in^5^; Fig. 4 in^12^; Supplement Fig. S2). The three stages are characterized by: Stage 1, social aggression including biting the mirror (described as mouth-fighting) typically within 2 days of mirror presentation; Stage 2, atypical behaviours (apparent C-testing) towards the mirror reflection – primarily between 3 and 5 days of mirror presentation; and Stage 3, gazing behaviours toward the mirror reflection – from days 3 to 5 onwards^5,12^. Comparable behavioural stages have been reported in other taxa considered capable of MSR (e.g^3,6,8)^, with Stage 3 traditionally being recognised as the point at which animals have recognised the self in the mirror and can now potentially pass the mark-test^5,12^. Consequently, previous MSR studies have only conducted the mark-test after 3 to 14 days of mirror exposure^3,5,8,10,12^. In past cleaner fish studies, these three behavioural stages appeared to overlap within individuals in the days after first exposure to a mirror (see^5,12^, Supplement Fig. S2). As we now understand, these behaviours are occurring post-MSR and are not related to achieving MSR. We therefore undertook a detailed re-examination of these behavioural stages, predicting that if these behaviours reflect true aggression, C-testing behaviour, and self-directed behaviours, they will occur in sequence and not overlap prior to the establishment of MSR.

### Experimental procedure

A high-quality mirror (45 cm x 28 cm) was secured on the outside of each glass experimental tank (*n* = 9). Mirrors were covered with a white plastic sheet while fish acclimatized to the tank, prior to the start of the experiment. A square rock (5 cm x 5 cm x 10 cm) was placed in the corner of each tank to provide substrate for cleaner fish to attempt to remove (via scraping) the mark placed on their throat (as per^5)^. All fish were placed in eugenol solution (0.1 ml / 500 ml, 10 min) (FA100; Tanabe Pharmacy, Tokyo, Japan) to achieve anaesthesia. All fish (*n* = 9) were marked via subcutaneous injection on the throat with a brown coloured visual implant elastomer (VIE). Detailed descriptions of the procedure, including control experiments to demonstrate the appropriateness of this method, can be found in^5,12^. Prior to mirror exposure, a video-camera was placed in front of each experimental tank.

The morning after the VIE mark placement on the throat (from 09:00), each fish was recorded for 20 min, to ensure they were swimming and behaving normally. The mirror was then uncovered and remained visible throughout the next 5 days. Every day, fish were video recorded for 8 h between 9:00 to 17:00, to document their mirror related behaviours and any changes in behaviour pre- and post-MSR.

Video-recordings were analysed using the analytical software ELAN. Recordings of the first day were analysed for the full 8 h, when fish exhibited mark removal behaviours (i.e., throat scraping). The timing and duration of aggressive behaviours, behaviours a - d (Table S1), mouth-touching behaviours, proximity to mirror (< 5 cm) and atypical behaviours a - e (Table S2) were identified and analysed. In addition, we also reanalysed the behavioural observations previously made of cleaner fish in^12^, these included atypical behaviours (Table 2 in^12^ )exhibited during mirror exposure.

We investigated any potential differences between pre-MSR aggression and post-MSR mouth-touching behaviours. Specifically, during these two periods we recorded: (1) frequency of caudal fin waving/5 sec up to 10 times per fish; (2) speed of “drifting down” events (distance in cm/ 5 s) up to 10 times per fish, and (3) swimming speed/ 10 s up to 6 times per fish. Swimming speed was measured when fish were swimming freely in the tank and not when foraging or reacting towards the mirror. We also confirmed whether cleaner fish exhibit shrimp lifting and dropping behaviour in the absence of a mirror, by video recording the activity of 5 mirror naïve cleaner fish, in a tank without a mirror, with access to a piece of shrimp for 2 h.

### Statistical analyses

In all experiments, fish behaviours in each test were analysed from video recordings. Several behaviours are descriptive in nature, like the distinct phases before cleaner fish scraped their throats. Data are summarized as medians and ranges in the text, while the raw data are shown in Fig. 1. All statistical analyses were conducted using R 4.1.1. For all models, we transformed data to successfully meet model assumptions, as confirmed by Shapiro-Wilk normality tests and Bartlett tests of homogeneity of variances being NS.

In a first set of tests shown in Fig. 2, we analysed three behaviours that increased in frequency after MSR. We first tested the frequency at which cleaner fish touched the mirror with their mouths with a linear mixed model fit by REML (LMM), with day as the main effect and cleaner fish identity as a random effect, using the formula, log (mouth touch + 0.05) ~ day + (1/id). We next tested for variation in the frequency of atypical swimming patterns in front of the mirror (‘self-directed behaviour’). Such behaviours did not occur pre-MSR or on day 1. We had to remove one outlier data point for one individual for day 5 because its residual was outside the distribution of the other data points, despite fitting the general trend that self directed behaviour was highest on day 5. With the removal, we were able to run a Bayesian linear mixed model fit by REML (Bayesian LMM), with day as the main effect and cleaner fish identity as a random effect, using the formula, self-directing ~ day + (1/id). A third analysis assessed the time that cleaner fish spent close or away from the mirror. We ran a linear mixed model fit by REML (LMM), with day as the main effect and cleaner fish identity as a random effect, using the formula, log (‘checking’ + 0.75) ~ day + (1/id). For all three analyses, we calculated Tukey contrasts as post hoc tests to identify which days differed significantly from each other.

Three additional analyses were conducted, again comparing cleaner fish behaviours across days (see Fig. 3). Specifically, the frequency of caudal fin beats when cleaner fish touched the mirror was analysed with a linear mixed model fit by REML (LMM), with day as the main effect and cleaner fish identity as a random effect, using the formula, log(beat frequency + 0.35) ~ day + (1/id), followed by Tukey post hoc test to identify which days differed significantly from each other. Next, how far individuals sink while touching the mirror with the mouth was analysed with a linear mixed model fit by REML (LMM), with day as the main effect and cleaner fish identity as a random effect, using the formula, log(sinking distance + 2) ~ day + (1/id), followed by Tukey post hoc test to identify which days differed significantly from each other. For this analysis we merged days 3 and 4 as there were only few individuals (3 + 2) that showed mirror interactions. Finally, average individual swimming speed was analysed with a linear mixed model (LMM), with day as the main effect and cleaner fish identity as a random effect, using the formula, log(swimming speed) ~ day + (1/id), followed by emmeans to identify which days differed significantly from each other. For this last analysis we had to exclude three data points with positive values (individuals moved up rather than stay put or moved down) to meet model assumptions.

## Supplementary Information

Below is the link to the electronic supplementary material.

Supplementary Material 1

Supplementary Material 2

Supplementary Material 3

Supplementary Material 4

Supplementary Material 5

Supplementary Material 6

Supplementary Material 7

Supplementary Material 8

Supplementary Material 9

Supplementary Material 10

## Data Availability

All the data are accessible within the main text and electronic supplementary materials (ESM).
