# Supplementary material for: Rapid self-recognition ability in the cleaner fish
Source: Sci Rep. 2025 Nov 25;15:41882. doi: 10.1038/s41598-025-25837-0 (PMC12647748; doi:10.1038/s41598-025-25837-0)
Supplement: Supplementary file 8 — Supplementary Material 8 [file 41598_2025_25837_MOESM8_ESM.docx]

Supplementary Materials for

**Rapid self-recognition ability in the cleaner fish**

Authors: Shumpei Sogawa^1^*, Taiga Kobayashi^1^, Redouan Bshary^2^, Will Sowersby^1,3^, Satoshi Awata^1^, Naoki Kubo^1^, Yuta Nakai^1^, Masanori Kohda^1^*

Corresponding author: [a10se013@yahoo.co.jp](mailto:a10se013@yahoo.co.jp)

**The PDF file includes:**

Figs. S1 to S2

**Other Supplementary Materials for this manuscript include the following:**

Movies S1 to S5

Data S1

Fig. S1.

Fig. S2. Frequency of caudal fin waving (A), distance falling down mirror (B) and swimming speed (C) in 8 individuals, originally reported in Kohda et al. 2022 (*11*). (A) LMM, *df* = 7, χ2 = 4.86, p = 0.30; (B) LMM, *df* = 7, χ2 = 6.79, p = 0.15; (C) LMM, *df* = 7, χ2 = 17.16, P < 0.01. Superscript labels a and b denote statistically significant differences.

Fig. S2.

Fig. S1. Apparent aggression (red line), frequency of apparent C-testing (green bar) and observing self-image near the mirror (blue line) observed in 7 cleaner fish, re-examined data from Kohda et al. 2019 (*4*). The three behavioural parameters typically overlap in an individual.

The red broken line in fish #2 is due to missing of the video of 3^rd^ day.


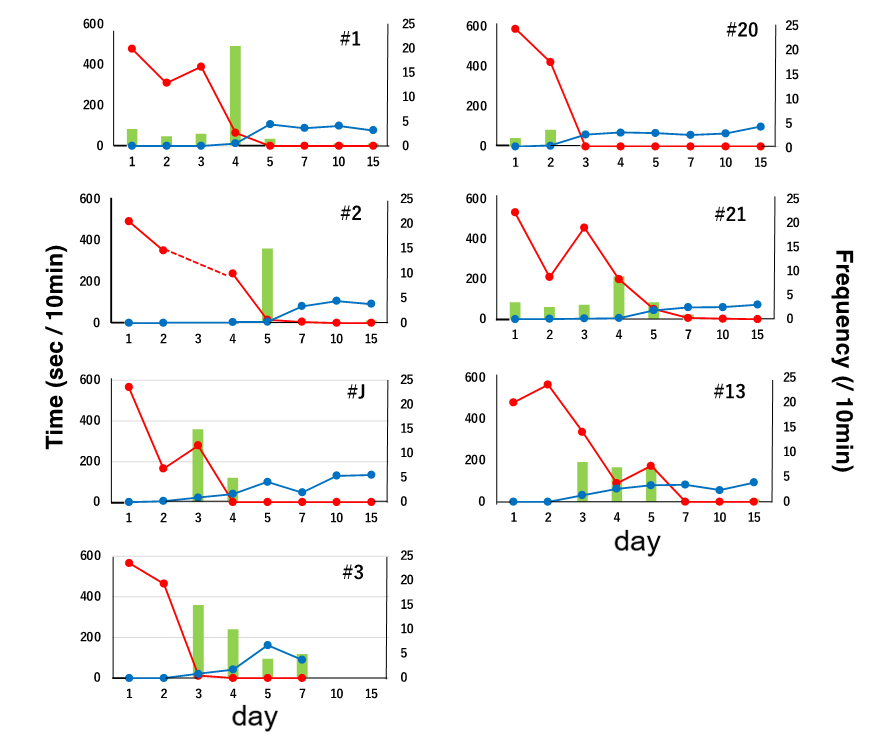


Movie S1 a, b.

The gazing behaviour toward the mirror while rapidly swimming away.

Movie S2.

The pre-MSR aggressive behaviour toward the mirror.

Movie S3.

The post-MSR mouth-touching behaviour toward the mirror.

Movie S4 a, b.

The dynamic and quick motions, such as rapid swimming along and against the mirror.

Movie S5.

The behaviour of observing sinking food reflected in a mirror.

Data S1. (separate file)

The Excel data for all figures (Fig. 1 to 3 and S1 to S2) and tables (Table S1 a, b).
